# Balancing Maternal and Fetal Well-Being: Ethical Complexities in Acute Psychosis Management During Pregnancy

**DOI:** 10.7759/cureus.82424

**Published:** 2025-04-17

**Authors:** Yarden Segal, Victoria Singh, Omar Nafeh, Meena Alzamani, Sasidhar Gunturu

**Affiliations:** 1 Psychiatry, BronxCare Health System, New York, USA; 2 Psychiatry, American University of the Caribbean School of Medicine, Cupecoy, SXM

**Keywords:** antipsychotics during pregnancy, antipsychotic use, ethical considerations, ethical dilemmas, maternal-fetal outcomes, maternal-fetal risks, medical ethical issues, multidisciplinary approach, peripartum psychosis, post-partum psychosis

## Abstract

While pregnancy is a time of well-being and happiness for most, it is a time of increased vulnerability to psychiatric illness for some. Women with psychiatric histories are more vulnerable to mood symptoms and psychosis during the peri- and post-partum period. These have detrimental effects both for the mother and the offspring. Maternal suicide risk, self-harming behaviors, and psychosis are increased compared to the general population. This presents both a psychiatric and obstetric emergency, with implicit clinical and ethical challenges. We present a 33-week pregnant female patient with a past psychiatric history of bipolar I disorder who was brought in by police after assaulting bystanders on the street. She provided a false name and had an alleged history of over 19 hospitalizations in the past year. She initially presented with catatonia, later observed to be aggressive and disorganized. She attempted to self-abort by hitting herself, throwing herself on the floor, and putting lotion in her vagina to help the baby “slide out”. The patient was treated with haloperidol, clozapine, and fluoxetine. This case presents the ethical implications of treating pregnant women with acute psychosis and the balance between beneficence, nonmaleficence, autonomy, and justice in an inpatient setting. These ethical dilemmas arise when the physician’s obligations to the mother and fetus diverge.

## Introduction

While pregnancy is often a time of well-being and positive emotional experiences, it can also represent a period of heightened vulnerability to psychiatric illness for some individuals. Women with pre-existing psychiatric histories are particularly at increased risk for mood disturbances and psychosis during the peri- and post-partum periods. Such episodes can have significant adverse consequences for both the mother and her offspring [1].

Women with a history of severe psychiatric illness are at elevated risk of relapse during the perinatal period, with postpartum relapse rates reported between 28% and 46%, particularly among those with bipolar or schizoaffective disorders. Several biological, psychological, and social risk factors have been implicated in perinatal psychosis, including hormonal fluctuations, sleep deprivation, discontinuation of psychiatric medications, stress, and a prior history of postpartum or affective psychosis. Additionally, the presence of positive, disorganization, and manic symptoms in the two years preceding pregnancy is associated with a significantly increased risk of perinatal relapse [2,3].

Psychosis refers to a cluster of severe symptoms such as hallucinations, delusions, disorganized thinking, and behavioral disturbances that can significantly impair functioning and may arise as a complication during pregnancy [4]. In the United States, the national prevalence of psychosis at the time of delivery was reported to be 698.76 per 100,000 hospitalizations in 2018 [3]. Those with a psychiatric history face heightened vulnerability to mood symptoms and psychosis during the peri- and post-partum phases, due to both physiological changes and high rates of treatment discontinuation.

Psychosis in pregnancy has been associated with poor outcomes for both mother and fetus, such as increased cesarean delivery, longer length of hospital stays, induction of labor, antepartum hemorrhage, placental abruption, postpartum hemorrhage, preterm delivery, and premature rupture of membranes [3]. Furthermore, untreated psychotic disorder in pregnant women has been associated with suicidal ideation, violent or homicidal ideation, or even infanticide [4]. Therefore, this presents as not only a psychiatric emergency but also an obstetric emergency, with implicit clinical and ethical challenges. 

We report a case involving a woman diagnosed with bipolar disorder who presented to our care in an acutely decompensated psychotic state at 33 weeks of pregnancy. In this report, we review her challenging presentation, focusing specifically on the ethical dilemmas that arise when the physician’s obligations to the mother and the fetus diverge regarding the management of an antepartum psychotic patient and comparing it to available literature, reinforcing the need for more collaborative care.

## Case presentation

A 26-year-old Hispanic woman was brought to the Emergency Department (ED) by the police after she assaulted bystanders on the street. In the ED, she provided a false name and was found to be 33 weeks pregnant, as confirmed by ultrasound. Urine toxicology was positive for cannabinoids and cocaine. She was diagnosed with latent syphilis, evidenced by a reactive rapid plasma reagin (RPR) test. Given the potential neuropsychiatric effects of untreated syphilis, this may have contributed to or exacerbated her current mental health presentation.

On admission, she exhibited catatonic symptoms including mutism, rigidity, staring, and mild psychomotor retardation. The following day, she displayed contrasting behavior characterized by pacing, frequent crying, and mood lability. She was diagnosed with schizoaffective disorder, bipolar type, confirmed based on her past medical history, prior hospital records, and symptom presentation upon admission.

Her psychiatric history, as per the Psychiatric Services and Clinical Knowledge Enhancement System (PSYCKES), a Health Insurance Portability and Accountability Act (HIPAA)-compliant web-based platform developed by the New York State Office of Mental Health, revealed a diagnosis of Bipolar I Disorder. She had more than eight inpatient hospitalizations in the preceding year and over 20 visits to the ED. Records from previous admissions indicated trials with various medications, including Depakote, Lithium, Haldol, Zyprexa, and Risperidone.

The patient seemed unaware of her pregnancy, expressing confusion and attempting to self-induce labor. In an attempt to self-abort, she ingested non-food items and placed foreign objects in her vagina. She filed a 72-hour letter requesting discharge and was legally retained until the birth of a child. Initially, she refused all medications, and Treatment Over Objection (TOO) was granted.

During her inpatient psychiatric hospitalization, the patient presented with disorganized thought processes, auditory hallucinations, paranoid delusions, and severely impaired insight. She demonstrated poor impulse control, verbal aggression, and required 1:1 supervision due to attempts to leave the unit against medical advice. Initial pharmacologic management included haloperidol 5 mg orally twice daily for acute psychosis and 20 mg fluoxetine once daily for comorbid anxiety and depressive symptoms.

Despite adherence to her medication regimen, the patient’s psychotic symptoms persisted, and she remained emotionally labile and disorganized. This led to the addition of olanzapine, titrated from 5 mg to 30 mg daily. However, her response remained minimal, consistent with a long-standing psychiatric history marked by multiple hospitalizations and poor responsiveness to standard antipsychotic treatments.

Clozapine was initiated due to treatment-refractory schizoaffective disorder, in accordance with evidence-based guidelines for patients who fail to respond to at least two adequate antipsychotic trials. Prior to initiation, appropriate baseline laboratory assessments were completed, and absolute neutrophil count (ANC) monitoring was established. During the course of treatment, the patient demonstrated gradual improvement in affect, reduction in hallucinations, and increased coherence in thought processes. While she remained guarded and insight was limited, her agitation subsided significantly, and she no longer displayed aggressive or impulsive behaviors.

Following delivery, the infant was medically stable without complications. Due to ongoing concerns about the patient's psychiatric stability, non-adherence, and limited capacity to care for the newborn, custody was transferred to the maternal grandmother through the Administration for Children’s Services (ACS). Legal proceedings formalized the temporary kinship arrangement.

At discharge, the patient was calm, cooperative, and no longer exhibiting overt psychotic symptoms. However, she continued to lack full insight into her illness. She was discharged on haloperidol decanoate 200 mg IM monthly, clozapine 400 mg twice daily, and fluoxetine 40 mg daily, and enrolled in an Assisted Outpatient Treatment (AOT) program with structured follow-up and outpatient psychiatric care.

## Discussion

In this case, the patient presented with acute psychosis during pregnancy in the context of a longstanding history of severe mental illness, including multiple psychiatric hospitalizations and poor medication adherence. Her presentation underscores the heightened risk of relapses and symptom exacerbation in pregnant individuals with pre-existing psychiatric conditions. One study revealed that 12% of pregnant women required psychiatric hospitalization, and 10% had to seek care in a psychiatric emergency department while pregnant [5]. This underscores the urgency of examining mental health issues during pregnancy to provide comprehensive perinatal care. Women with bipolar I disorder experience a high risk of perinatal mood episodes, with approximately 23% experiencing an episode during pregnancy and 52% during the postpartum period, highlighting the particularly vulnerable nature of the postpartum phase for the recurrence of affective or psychotic symptoms [6].

Current United States guidelines, including those from the American College of Obstetricians and Gynecologists and the United States Preventive Services Task Force, recommend universal screening for depression and anxiety throughout pregnancy and the postpartum period using validated tools [7]. However, despite the serious consequences of conditions such as psychosis and bipolar disorder, routine screening for these severe mental illnesses is not yet universally mandated.

The use of antipsychotic medications during pregnancy requires a careful, individualized risk-benefit analysis. Clozapine, in particular, is typically reserved for treatment-resistant cases due to potential risks such as metabolic disturbances, sedation, and, rarely, agranulocytosis. Although data on clozapine use in pregnancy are limited, current evidence does not indicate a high risk of teratogenicity. Nonetheless, close neonatal monitoring is recommended due to the potential for withdrawal symptoms or toxicity.

Effective management of perinatal psychosis requires a tailored approach that ensures both maternal stability and fetal safety. In this case, haloperidol and olanzapine were ineffective despite adequate dosing, leading to the initiation of clozapine under strict monitoring, which resulted in significant symptom improvement. Clozapine, typically reserved for treatment-resistant cases due to risks like sedation, metabolic effects, and rare agranulocytosis, may also increase the risk of gestational diabetes, floppy infant syndrome, and neonatal seizures. Though data is limited, these risks should be discussed, and close neonatal monitoring is essential. This case highlights the importance of individualized treatment when standard therapies fail [8]. Psychosis risk in pregnancy is influenced by several prenatal and perinatal factors. These include young or advanced parental age, family history of psychiatric illness, especially maternal psychosis, and maternal infections. Obstetric complications such as hypoxia, hypertension, and premature rupture of membranes also contribute. Low birthweight, congenital malformations, and poor fetal growth increase risk. Environmental factors like famine, maternal stress, and inadequate antenatal care are also linked [9]. 

In women with mental illness, the risk of developing psychosis during pregnancy is significant, but they are also more prone to encountering sexually transmitted infections, gynecological disorders, and reproductive health cancers. These factors can significantly impact fertility and pregnancy outcomes [10]. Adding to these complexities is the challenge of limited access to cervical screenings and preventive contraceptives compared to women without mental disorders. Additionally, inadequate access to comprehensive family planning services contributes to a higher incidence of unplanned pregnancies [10]. A study has suggested that women with mental illness, particularly bipolar disorder, demonstrate an increased inclination toward engaging in risky sexual behaviors with multiple partners, heightening their vulnerability to sexually transmitted infections and unplanned pregnancies [11]. This risk is further amplified when there is a concurrent substance use disorder. Research indicates that this subgroup is more likely to have multiple sexual partners and may resort to transactional sex for substances [11]. These findings are relevant to the patient in this case report who presents with a substance use disorder, specifically cocaine use, and indicates a likelihood of her involvement in risky sexual behaviors or engaging in transactional sex. Consequently, this elevated her risk of experiencing an unplanned and unwanted pregnancy.

A significant disparity exists in the field of sexual and reproductive health when it comes to women with severe mental illness (SMI) [10]. Consequently, healthcare providers bear the responsibility of delivering comprehensive education to individuals with SMI who are contemplating pregnancy and offering guidance to those who encounter unplanned pregnancies. It's noteworthy that younger patients with SMI tend to have lower levels of knowledge regarding sexual and reproductive health when compared to their mentally healthy counterparts [10]. This knowledge gap often persists over time. To address this issue effectively, a close collaboration between obstetrics and gynecology and psychiatric services is of utmost importance to ensure that every patient is well-informed [12]. Routine care must include discussions about sexual decision-making, safety, preferred contraception methods, and family planning. Additionally, healthcare providers should assess cervical screening and human papillomavirus (HPV) vaccination uptake. Regrettably, these aspects are often overlooked, especially within the domain of mental health services [10].

There is a strong and positive correlation between self-harm incidents among pregnant women and psychosis, often coinciding with concurrent substance abuse. In a study encompassing 420 women with SMI, 33 reported a total of 52 self-harm events during their pregnancies [13]. Among these reported events, 33 were attributed to hallucinations. It remains unclear from this study whether the patients adhered to their treatment plans during these episodes of self-harm. However, it is crucial to highlight that 78.6% of participants discontinued their medication due to concerns about potential teratogenic risks to their unborn child [13].

Untreated psychosis during pregnancy poses substantial risks to both the expectant mother and the unborn child, as shown in Table 1 [14]. An analysis covering 23,507,597 delivery hospitalizations between 2007 and 2012 revealed that the presence of psychosis in pregnant women was associated with a range of adverse outcomes [3]. These included cesarean delivery, extended hospital stays, the need for labor induction, antepartum hemorrhage, placental abruption, postpartum hemorrhage, preterm delivery, and premature rupture of membranes. Additionally, infants born to mothers experiencing antepartum psychosis faced an elevated risk of stillbirth, poor fetal growth, fetal distress, and fetal abnormalities. The risk of low birth weight significantly rises among mothers with schizophrenia, especially those who experience a relapse of their illness during pregnancy [3].

**Table 1:** Potential risks of untreated psychosis in pregnancy Reference: Friedman et al., 2018 [14]

Risks	Details
Suicide	Elevated risk, particularly in the postpartum period
Intentional and unintentional self-harm	Includes dangerous behaviors and accidents due to impaired judgment
Infanticide and/or child abuse	Documented in severe untreated psychotic episodes
Poor prenatal and postnatal care	Missed appointments, non-adherence to medical recommendations
Increased use of illicit substances	Often used as a coping mechanism, exacerbating psychiatric instability
Poor maternal outcomes	Cesarean delivery, extended hospital stays, induction, hemorrhage, abruption
Poor infant outcomes	Low birth weight, stillbirth, poor fetal growth, fetal distress, abnormalities
Impaired mother-infant bonding	Can result in attachment issues and long-term developmental impact

As healthcare professionals, it is imperative to uphold the foundational principles of medical ethics when caring for all patients. However, complex cases like the one at hand can render ethical treatment planning particularly challenging. This case presented several ethical dilemmas, beginning with the patient’s refusal of treatment. While respecting patient autonomy is essential, concerns regarding her capacity to make informed medical decisions led to the initiation of legal procedures such as TOO. Ethical dilemmas arose early in this case, beginning with the patient’s refusal of treatment. While autonomy is a core principle, concerns about her decision-making capacity led to the use of TOO, a court-authorized process for involuntary treatment when a patient poses a danger to self or others. This process varies by state; in New York, it is guided by the Mental Hygiene Law, New York State Office of Mental Health (NYS OMH) guidelines. The decision prioritized beneficence to prevent harm to both the patient and fetus, underscoring the complex balance between ethics and legal care [15]. In contrast, Hill et al. demonstrated that postpartum psychosis can be effectively managed within specialized Mother-Baby Units (MBUs), where all mothers in their study retained custody, and most infants developed normally [16]. Our case of antepartum psychosis, however, required involuntary inpatient treatment and led to temporary loss of custody, reflecting a critical gap in care infrastructure and protocols for managing psychosis during pregnancy.

The complexity of treatment planning is compounded not only by patient refusal but also by the concerns surrounding the available treatment options. Antipsychotic medications represent the established standard of care for managing psychotic disorders in pregnant women, encompassing conditions like bipolar disorder, schizophrenia, and other psychotic disorders [17]. Discontinuing antipsychotic treatment during pregnancy carries the potential for an increased risk of relapse. Opting for non-treatment would be more detrimental to both the mother and the fetus, as untreated psychosis typically leads to the deterioration and further decompensation of the mother's condition. When contemplating the initiation of treatment, it is imperative to thoroughly assess the ethical concern regarding potential adverse effects on the unborn child. Psychotropic medications can readily cross the placenta due to their small molecular size. Haloperidol, classified as Category C in pregnancy, indicates that risk cannot be definitively ruled out [17]. While there remains a general lack of adequate and controlled studies about the use of haloperidol during pregnancy, a single study identified two isolated cases of haloperidol use with other medications for hyperemesis gravidarum in the first trimester leading to infants born with severe limb reduction defects, although a causal relationship could not be established [17]. Notably, no reports of teratogenicity linked to haloperidol have surfaced during the second and third trimesters. While the fetal risk associated with haloperidol appears to be low, these considerations play a pivotal role in the decision-making process when determining the treatment plan for acutely psychotic pregnant patients.

Enhancing our understanding of the triggers of psychosis in pregnant and postpartum women can facilitate the identification of those at a heightened risk. To provide more effective care for acutely ill pregnant women, there is a pressing need for a standardized approach, which currently does not exist [18]. The absence of a standardized approach to care will continue to result in complications, such as adverse health outcomes for the fetus and an elevated risk of comorbidities in the mother. Notably, there are significant associations between maternal mental disorders and pre-term births [19]. It's essential to recognize that many of these complications can be exacerbated by the numerous comorbidities often accompanying mental illness. Further research is imperative to gain a more comprehensive understanding of the triggers for psychotic episodes during pregnancy and childbirth, the identification of women at risk, and the development of improved treatments for pregnant women experiencing mental illness [20].

## Conclusions

Physicians caring for acutely psychotic pregnant patients face complex clinical and ethical challenges, compounded by the lack of universally accepted, risk-free treatment protocols. Managing antepartum psychosis demands adherence to core medical ethics and interprofessional collaboration to ensure safe, compassionate care. While case reports and clinical experience offer insight, the absence of standardized, evidence-based guidelines highlights the urgent need for updated recommendations tailored to this population. A systematic review of current treatment protocols, alongside further research into risk factors and therapeutic strategies, would be valuable in improving outcomes for both mother and child. Strengthening partnerships between obstetric and psychiatric teams remains critical in optimizing care throughout the perinatal period.
